# Identification of Potential Genes for Benign Prostatic Hyperplasia and Prostate Cancer Susceptibility in Four X-chromosome Regions with High Frequency of Microvariant Alleles

**DOI:** 10.31557/APJCP.2020.21.8.2271

**Published:** 2020-08

**Authors:** Mohammed H Albujja, Safia A Messaoudi, Ramachandran Vasudevan, Saleh Al Ghamdi, Pei Pei Chong, Khairul Asri Ghani, Yazan Ranneh, Mohammed Alaidarous, Patimah Ismail

**Affiliations:** 1 *Department of* *Forensic Sciences, Faculty of Criminal Justice, Naif Arab University for Security Sciences, Riyadh, Saudi Arabia. *; 2 *Department of Biomedical Sciences, Faculty of Medicine and Health Sciences, Universiti Putra Malaysia, Serdang, Selangor, Malaysia. *; 3 *Research Center, King Fahad Medical City, Riyadh, Kingdom of Saudi Arabia. *; 4 *School of Biosciences, Faculty of Health and Medical Sciences, Taylor University, Subang Jaya, Selangor, Malaysia. *; 5 *Department of Surgery, Faculty of Medicine and Health Sciences, Universiti Putra Malaysia, Serdang, Selangor Malaysia. *; 6 *Department of Technology and Natural Resources, Faculty of Applied Sciences and Technology, Universiti Tun Hussein Onn Malaysia, Parit Raja, Batu Pahat Johor, Malaysia.*; 7 *Department of Medical Laboratory Sciences, College of Applied Medical Sciences, Majmaah University, Riyadh, Saudi Arabia. *

**Keywords:** Benign Prostate Hyperplasia (BPH), Prostate cancer (PrCa, X-chromosome (ChrX)

## Abstract

**Background:**

The X-chromosome has been suggested to play a role in prostate cancer (PrCa) since epidemiological studies have provided evidence for an X-linked mode of inheritance for PrCa based on the higher relative risk among men who report an affected brother(s) as compared to those reporting an affected father. The aim of this study was to examine the potential association between the forensic STR markers located at four regions Xp22.31, Xq11.2-12, Xq26.2, and Xq28 and the risk of BPH and PrCa to confirm the impact of ChrX in the PrCa incidence. This may be helpful in the incorporation of *STRs* genetic variation in the early detection of men population at risk of developing PrCa.

**Methods:**

DNA samples from 92 patients and 156 healthy controls collected from two medical centers in Riyadh, Saudi Arabia were analyzed for four regions located at X-chromosome using the Investigator^®^ Argus X-12 QS Kit.

**Results:**

The results demonstrated that microvariant alleles of (DXS7132, DXS10146, HPRTB, DXS10134, and DXS10135) are overrepresented in the BPH group (p < 0.00001). Allele 28 of DXS10135 and allele 15 of DXS7423 could have a protective effect, OR 0.229 (95%CI, 0.066-0.79); and OR 0.439 (95%CI, 0.208-0.925). On the other hand, patients carrying allele 23 of DXS10079 and allele 26 of DXS10148 presented an increased risk to PrCa OR 4.714 (95%CI, 3.604-6.166).

**Conclusion:**

The results are in concordance with the involvement of the X chromosome in PrCa and BPH development. STR allele studies may add further information from the definition of a genetic profile of PrCa resistance or susceptibility. As *TBL1, AR, LDOC1*, and* RPL10* genes are located at regions *Xp22.31, Xq11.2-12, Xq26.2*, and *Xq28*, respectively, these genes could play an essential role in PrCa or BPH.

## Introduction

The prostate of humans is located at the base of the urinary bladder. It is considered as the source of three main causes of morbidity; benign prostatic hyperplasia (BPH), prostate cancer (PrCa) and prostatitis. BPH is a nonmalignant enlargement of the prostate gland and refers to the stromal and glandular epithelial hyperplasia that occurs in the transition zone of the prostate; even though, BPH is not generally considered to be a precursor lesion to prostate cancer (Aaron et al., 2016).

BPH is a very common disease among elderly men. In general, its prevalence rate increases in men above the age of fifty. In the United States only, it has been estimated that the annually cost for this disease is about twelve million (Lee et al., 2016). The co-existence of PrCa in males who treated for BPH is between 3-20% (Yeboah and Hsing, 2016). Furthermore, when BPH is undetected at an early stage, cancer has a chance to develop and progress not only in size, but also in its capacity to interfere and perturb neighboring cells. There are evidences suggesting that BPH and prostatic adenocarcinoma share common predisposing factors (Guess, 2001; Yeboah and Hsing, 2016). There is no doubt that patients with BPH represent a unique category of patients with special symptoms, which need to be identified.

Even though the mechanistic basis for the initiation and progression of BPH from asymptomatic to symptomatic remains unclear, aging, genetic factors, hormonal changes and lifestyles are considered as potential causes which may attributed to the overgrowth of smooth muscle tissue and glandular epithelial tissue in the prostate (Aaron et al., 2016).

The androgen receptor (AR) signaling axis plays a key role in androgen-dependent PrCa (Filella el al., 2018). It has been established that removal of androgens makes the PrCa growth rapidly decline with significant clinical response (Rubin and Marzo, 2004). In addition, it plays a key role in development of BPH, because, the polymorphic CAG repeat in the AR can alter transactivation of androgen-responsive genes and potentially influence BPH risk. Therefore, Aaron et al. declared that the detailed understanding of the pathways that lead to the genesis of BPH nodules would assist in the design of better or complementary therapies (Aaron et al., 2016).

From the observations in clinical genetics, we know that persons, who share a very rare genetic feature, can be combined in a common pedigree (Szibor, 2007). Also, several genetic epidemiological studies confirmed the higher risk of PrCa in men with affected brother than with affected father (Bratt et al., 1999; Kral et al., 2011); supported by the fact that father and son would not share any X-chromosomal alleles identical by descent (ibd). In contrast, brothers share a given maternal X-chromosomal allele with a probability of 0.5 (Szibor, 2007). 

The X-chromosome (ChrX) is 155 million base pairs (Mb) long and carries approximately 1,250 known genes. That X-linked or recessive mode of inheritance for PrCa is characteristic of genetic traits known to be on the ChrX and passed from maternal grandfather to mother and then to offspring. The abovementioned short casuistic is aimed at demonstrating the power of ChrX markers in male specific genetic diseases in general and in particular BPH.

Short tandem repeats (STRs) are regions in human genome with two to six bp repeat units in length. These repeats are variable in number among people, which make them effective for human identification applications. Kimpton et al., (1995) demonstrated that the STRs used in forensic field can also be used for screening genome to find loci related to diseases, due to their highly polymorphic nature, abundant presence, and wide distribution throughout the human genome (Willems et al., 2014).

STRs have been used for high-resolution human genome mapping and in population studies, it is also associated with up to 40 human monogenic diseases such as oculopharyngeal muscular dystrophy. Therefore, STRs are utilized in the indirect preimplantation genetic testing for monogenic gene defects (PGT-M), benefiting from the polymorphic STRs linked to the causal gene of disease. Moreover, some complex diseases such as cystic fibrosis and asthma are associated with certain STRs (Moya et al., 2018). These alleles have a Linkage disequilibrium (LD) with that diseases which refers to the nonrandom association of alleles at different loci on the same chromosome; LD has also been used to localize genes that are involved in monogenic disease (Jorde, 2000).

There are a considerable studies have been conducted to investigate the association between CAG and or GGC repeats located in Xq11.2, and incidence of BPH and PrCa (Alptekin et al., 2012; Biolchi et al., 2012; Cunningham et al., 2007; Giovannucci et al., 1997; Gsur et al., 2002; Gu et al., 2012; Gudmundsson et al., 2008; Lee et al., 2016; Neto et al., 2008; D. Riley and Krieger, 2001). Riley and Krieger mentioned that further study of STRs closely linked to PrCa-predisposing genes may provide new tools for understanding cancer progression and predisposition. Due to evidence that some prostate diseases are a risk factor for PrCa, some of these STRs may also be valuable in association research for these diseases (Riley and Krieger, 2002).

In the present study, we studied forensic STR markers located at four regions *Xp22.31, Xq11.2-12, Xq26.2 *and *Xq28* to search for BPH and PrCa risk gene loci based on the confirmed contribution of ChrX genes to PrCa incidence (Agalliu et al., 2010; Bochum et al., 2002; Kral et al., 2011; Lange et al., 1999; Xu et al., 1998). This may help to incorporate STRs genetic variation in the early detection of men population at risk of developing PrCa. Moreover, studies on BPH are limited and there is a pressing need for new approaches.

## Materials and Methods

*Sampling*

A case-control study was conducted to analyze X-STR associated with BPH and PrCa incidence in a Saudi male cohort. A total of 248 subjects were recruited comprising 92 patients with histologically verified prostate biopsy samples (44 PrCa and 48 BPH), and 156 healthy controls after verification of their health status from medical reports.

All participants were Saudis living in Riyadh. Samples were collected from two medical centers in Riyadh, The King Fahd Medical City (KFMC), and King Abdulaziz Medical City (KAMC). PrCa patients were selected from the registries of the Oncology Departments, which contain case report information about the clinical status and follow-up of patients.

Ethics clearance was acquired from a local ethics committee in KFMC (Reference number: 16-382) and an ethics committee in KAMC (Reference number: RC17/260/R). Protocols and informed consent were reviewed and approved. The study was clarified to potential participants and informed consent was obtained prior to sample collection.

From all volunteers, 2 mL whole blood was collected in EDTA vacutainer tubes by venous puncture. Following collection, samples were stored in a freezer at −80^o^C, until use.

*Genomic DNA extraction*

A ReliaPrep™ Blood gDNA Miniprep System (Promega Corporation, Madison, WI, USA) was used to extract genomic DNA. The DNA extraction method followed the protocols suggested by the manufacturer (Promega, 2012).

*Genomic DNA quantification*

All extracted genomic DNA were quantified using the Quantifiler^®^ Duo DNA Quantification Kit (Thermo Fisher Scientific Inc.; Waltham, MA, USA; Cat. #4387746). DNA quantification methods followed the protocols suggested by the manufacturer (Thermo Fisher Scientific, 2014).

*DNA amplification and fragment detection*

The twelve X-STR loci were simultaneously amplified by multiplex PCR using the Investigator^®^ Argus X-12 QS kit (Qiagen, Hilden, Germany). These STRs are distributed as four closely linked pairs over the entire ChrX, and for practical reasons they are assigned to four linkage groups (LG) 1–4. The genetic distance within the STR pairs is assumed to be <1 cM, whereas the pair to pair space is about 50 cM or more (Becker et al., 2008). Multiplex PCR was performed on an Applied Biosystems Veriti Thermal Cycler (Thermo Fisher Scientific Inc.) in a 13 μL reaction volume containing 12 µL Hi-Di formamide with appropriate 0.5 µL size standard and 1 µL amplified PCR product. The samples were denatured for 3 minutes at 95^o^C and snap chilled on ice before loading onto the instrument. Amplified fragments were separated by capillary electrophoresis using 3500 Genetic Analyzer (Thermo Fisher Scientific Inc.), following the manufacturer’s instructions and size separated with internal lane size standard BTO-5 were as recommended by the supplier (Qiagen, Hilden, Germany).

Alleles were assigned according to the International Society of Forensic Genetics (ISFG) guidelines for forensic STR by comparing to the reference allelic ladder included in the kit using GeneMapper ID-X software v.5.1 (Thermo Fisher Scientific Inc.). A peak detection threshold of 50 RFUs was used for allele designation.

*Statistical analysis*

All statistical analysis was performed using SPSS version 22.0 software (Statistical Package for Social Sciences, Chicago, IL). Chi-square analysis was used to compare categorical variables. A 5% level of significance was used in the analysis. The odds ratio (OR) and its 95% confidence interval (CI) were calculated as a measure of the association between alleles and BPH/PrCa risk. Continuous variables were expressed as mean ± standard deviation (SD) and were compared with the Student’s t-test (2-tailed) for comparison of PrCa and BPH groups based on ages, non-parametric rank-based Man-Whitney U to compare between two independent groups, and the Kruskal–Wallis test for independent multigroup comparisons. The strength of association between ChrX loci alleles and PrCa was estimated by odds ratio and 95% confidence intervals. *p*<0.05 was considered to be statistically significant.

Allele frequencies and haplotype frequencies of the respective STR pairs were estimated for each locus using StatsX v2.0 software (Lang et al., 2019). Linkage disequilibrium (LD) between pair of loci were estimated with STRAF - a convenient online tool for STR data evaluation in forensic genetics (Gouy and Zieger, 2017).

## Results

*Intermediate alleles (IA)*

**Figure 1:**
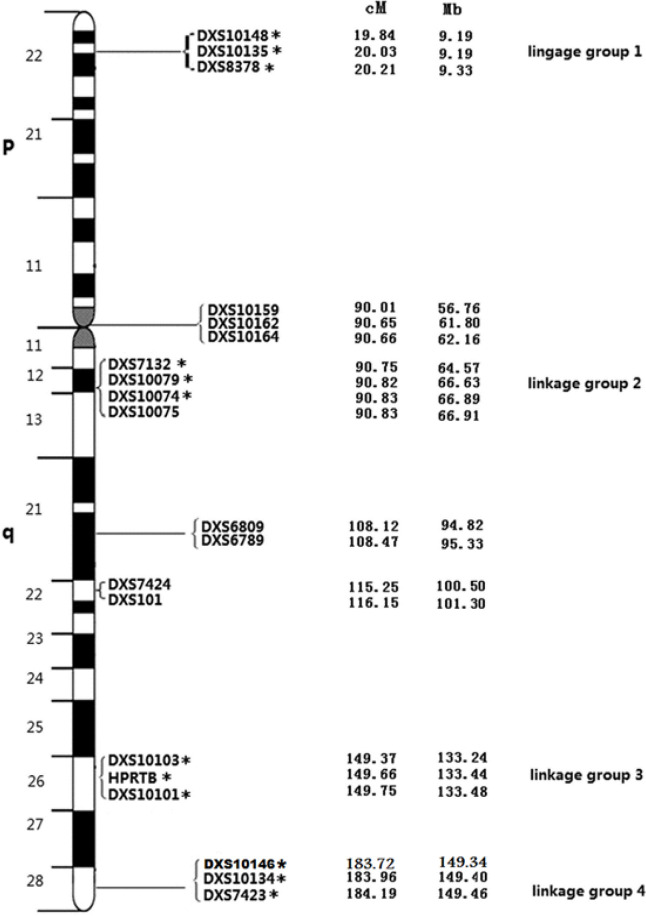
The Ideogram of the X-Chromosome Describes the Genetic Positions of the 19 X-STR Loci and Their Physical Location in the X Chromosome. Distances from the p-telomere are shown in cM and Mb; *, indicate the 12 X-STR loci that are shared with the Investigator Argus X-12 QS kit (Qiagen, Hilden, Germany). Adopted from (Qiagen, 2015).

Our study focuses on STR forensic markers at regions Xp22.31, Xq11.2-12, Xq26.2 and Xq28 with the goal of identifying candidate BPH or PrCa risk loci on ChrX as shown in Figure 1. The microvariant alleles (12.2, 13.2, 14.2) of DXS7132 locus, (34.2, 35.3, 36.3) of DXS10134 locus, 16.1 of DXS10074 locus, 22.2 of DXS10135 locus, 37.2 of DXS10146 locus, 21.2 of DXS10079 locus, (18.2, 41.1) of DXS10148 locus, and (11.2 and 12.2) of HPRTB locus were observed in patients with BPH in current study, while they did not recorded either in the four subpopulations of the US population or Investigator Argus X-12 QS kit allelic ladder (Diegoli et al., 2011; Qiagen, 2015).

Some microvariant alleles like 20.1 and 35.1 of DXS10148 locus were found in current study population and in some of the US subpopulations; while, the alleles 15.3 and 16.3 of DXS7132 locus and three microvariant alleles 21.1, 31.1, and 32.2 of DXS10148 locus exist only in the US population and do not exist either in the current study population (Diegoli et al., 2011).

We found that in the group of BPH patients under current study, microvariant alleles (rare alleles that are observed in the human population and that fall in between alleles with full repeat units by containing an incomplete repeat unit) (Butler, 2011) for (DXS7132, DXS10146, HPRTB, DXS10134 and DXS10135) loci are overrepresented (21 of 48 in the BPH group in comparison to none of 156 in the healthy control group; *p*<0.00001), suggesting that men carrying (DXS7132, DXS10146, HPRTB, DXS10134 and DXS10135) microvariant alleles are at increased risk for BPH.

For DXS10135 point-1-allele have revealed a 3 bp deletion within the downstream flanking region of the sequence 5’-AGAGAATAGAAAA(GAA/-)GAGAAGAGAAAA-3’ (deletion–insertion polymorphism in brackets) as characterized by Becker et al., (2008).

Allele 11.2 in HPRTB revealed a regular repeat motif and a common 2 bp deletion (AG) within the downstream flanking region of the sequence as shown in Table 1, confirmed by the sequencing results of Zalán et al., (2007).

At the DXS10134 locus parallel occurrence of point-3- allele (36.3) was found. The allele 36.3 contains an additional triple A insertion within the variable repeat giving the partial sequence (GAAA)3 AAA (GAAA)4 AAA (GAAA)4 AAA (GAAA)13–20. Allele sequences and number of alleles for DXS10134 were published by Becker et al., (2008).

Table 1 showing the sequencing data of loci motif and the microvariant alleles for DXS7132, DXS10146, HPRTB, DXS10134, DXS10135 and DXS10148 reported by previous studies (Becker et al., 2008; Bekada et al., 2010; Edelmann et al., 2008; Eun et al., 2010; Gomes et al., 2009; Zalán et al., 2007).

*Xp22.31, Xq11.2-12, Xq26.2 and Xq28 regions*

As DXS7132, DXS10146, HPRTB, DXS10134 and DXS10135 loci are located at regions Xp22.31, Xq11.2-12, Xq26 and Xq28, respectively, and in these loci the interalleles were overrepresented, we analyzed all loci presented in the Investigator^®^ Argus X-12 QS kit for four areas. No statistical differences were found for DXS8378, and DXS10074. However, we found a protective effect for alleles 28 and 20 of DXS10135 and men carrying these alleles present a lower risk of BPH or PrCa, OR 0.229 (95%CI, 066-0.794). Moreover, this protective effect was also found in men carrying allele 15 of DXS7423, OR 0.439 (95%CI, 0.208-0.925), as shown in Table 2. On the other hand, men with allele 23 of DXS10079 and allele 26 of DXS10148 showed a significantly higher risk to develop PrCa, OR 4.714 (95%CI, 3.604–6.166), see Table 2.

We further analyzed the combined protective or risk effect in individuals simultaneously carrying alleles [10, 21, 23.1] haplotype (a lineable combination of alleles at multiple loci that are transmitted together) of cluster 1 (DXS8378, DXS10135 and DXS10148) showed a higher frequency among patients with PrCa than among healthy controls. We found that they are overrepresented in the PrCa group (3 of 44 vs. none of 156 in normal control; *p*<0.001). While haplotypes [11, 30, 24.1] of cluster 1 and [19, 32.2, 12.2], [19, 30.2, 11.2] and [17, 30, 14] of cluster 3 (DXS10103, DXS10101 and HPRTB) had a higher frequency among patients with BPH than among controls. Also, [34, 14, 29] haplotype of cluster 4 (DXS10134, DXS7423 and DXS10146) had a higher frequency among patients with PrCa than among healthy controls. Therefore, men who have these specific haplotypes present a higher risk to develop PrCa or BPH, it is pertinent to note that three of four haplotypes which overexpressed in BPH patient comprised microvariant alleles, and five of seven significant haplotypes have microvariant allele, as shown in Table 3.

*Genetic linkage between X chromosome haplotypes and prostate tumor genes*

The linkage between STRs and diseases fall into two categories of relation; one individual STR or group of STRs may occupy a position close to a particular gene liable on a genetic disorder, and may therefore be used to follow the progress of the diseased gene within a family; it would not be possible to make any conclusions about the disease status of an individual in isolation of information about the family pedigree to identify the disease linked allele. More rarely, an STR may be directly responsible for a genetic disease like; Huntingtons disease, fragile X syndrome, and myotonic muscular dystrophy, or display an allele(s) directly associated with a disease within a given population (Kimpton et al., 1995). To find the disease related genes adjacent to the clusters in chromosome X which examined in this study, a network-based approach (UCSC Genome Browser tool suite) have been used to find and allocate these genes. This online website at (http://genome.ucsc.edu/), annotate genes, computes multiple alignments, predicts regulatory function and collects disease data, it has been utilized to seek these related genes to identify the most likely candidate genes which could have LD with clusters in the current study haplotypes, subsequently with its haplotypes. we hypothesized that the significant genetic polymorphic STRs biomarkers or haplotypes may be in a LD with tumor-related genes such as oncogenes or tumor suppressor genes. So, we inspected for the nearby genes to the significant haplotypes as listed in Table 3. 

We have observed two types of genes, over expressed genes in PrCa tissues in comparison with normal tissues which are associated with the existence of cancer, these genes make male more susceptible to PrCa, on the other hand, the tumor suppressor genes which have a lower or the lacked expression in PrCa tissues in comparison to normal prostate tissues, the absence of these genes are associated with the presence of cancer, or the aggressiveness or metastasis of cancer, while the presence of these genes make male less susceptible to PrCa.

These genes, either carcinogenic or tumor suppressor (protective) genes, may segregate with their physically adjacent microsatellites or haplotypes of linkage group as shown in the Table 3. Therefore, inherited together or passed to the next generation. This mechanism of inheritance makes these satellites an indirect genetic biomarker that refer to the susceptibility or resistance of subject to the corresponding disease.

**Table 1:** Sequencing Data of Loci Motif and of Microvariant Alleles

Locus	Sequenced Allele	Motif	Sequence structure of the repeat	Reference
DXS10135	20.1*	[AAGA]3 GAAAG [GAAA]20	5’-AGAGAATAGAAAA(GAA/-) GAGAAGAGAAAA-3’	(Becker et al., 2008)
HPRTB	11.2*	[AGAT]12*	2 bp deletion (AG); N50 ..GGGTG	(Zalán, Völgyi, Jung, & Ag, 2007)
DXS10134	36.3*	[GAAA]3 GAGA [GAAA]4 AA [GAAA] GAGA [GAAA]4 GAGA [GACAGA]3 [GAAA] GTAA [GAAA]3 AAA [GAAA]4 AAA [GAAA]15	(GAAA)3 AAA (GAAA)4 AAA (GAAA)4 AAA (GAAA)13–20	(Becker et al., 2008)
DXS7132	16.3	[TCTA]13	Pr1(22bp) 1bp (CTAT)14 CAT (CTAT)2 37bp (20bp)Pr2	(Gomes, Pereira, & Mayr, 2009)
DXS7132	17.3		Pr1(22bp) 1bp (CTAT)15 CAT (CTAT)2 37bp (20bp)Pr2	
DXS7132	18.3		Pr1(22bp) 1bp (CTAT)16 CAT (CTAT)2 37bp (20bp)Pr2	
DXS10146	39.2	[TTCC]3 T [TTCC]3 TTTC CTCCCTTCC [TTCC] [TCCC] TTCTTCTTTC [TTCC]2 TTTCTT [CTTT]2 CTTC [CTTT]10 T [CTTT]2	(TTCC)13 (CTTT)6 TT (CTTT)10;	(Edelmann, Hering, Augustin, & Szibor, 2008)
DXS10146	33.2		(TTCC)3 (CTTT)16 CT (CTTT)4	
DXS10148	20.1	[GGAA]4[AAGA]12[AAAG]4 N8 [AAGG]2	PF-[GGAA]4-[AAGA]11-A^a^ -[AAAG]3-N8-[AAGG]2–N104-PR	(Bekada, Benhamamouch, Menegon, Torre, & Robino, 2010)
DXS10148	18.1		(GGAA)4(AAGA)7A(AAGA)(AAAG)4-N8-(AAGG)2-N104	(Eun et al., 2010)
DXS10148	26.2		(GGAA)4(AAGA)14A(AAGA)A(AAGA)(AAAG)4-N8-(AAGG)2-N104	

^a^, Insertion giving rise to intermediate alleles; *, Microvariant alleles found in this study.

**Table 2:** X Chromosome Loci Allele Frequency Comparisons between Patients and Healthy Controls Based on Chi-square Test

Locus	Number of Repeats (Allele)	Patients		Healthy Controls		χ2	P	OR	95% CI for OR		Sig.
		No.	Freq.	No.	Freq.						
Benign Prostate Hyperplasia (BPH)											
DXS7132	14.2	2	0.042	0	0	6.564	0.01	4.391	3.406	5.661	IR
DXS7132	12.2	2	0.042	0	0	6.564	0.01	4.391	3.406	5.661	IR
DXS10134	36.3	3	0.063	0	0	9.896	0.002	4.467	3.453	5.778	IR
DXS10135	28	0	0	20	0.128	6.823	0.009	1.353	1.242	1.474	PRO
DXS10135	20.1	3	0.063	0	0	9.896	0.002	4.467	3.453	5.778	IR
DXS7423	15	11	0.229	63	0.404	4.845	0.028	0.439	0.208	0.925	PRO
DXS10146	38.2	2	0.042	0	0	6.564	0.01	4.391	3.406	5.661	IR
DXS10146	34.2	2	0.042	0	0	6.564	0.01	4.391	3.406	5.661	IR
DXS10079	17	5	0.104	5	0.032	4.095	0.043*	3.512	0.971	12.694	IR
HPRTB	12.2	3	0.063	0	0	9.896	0.002	4.467	3.453	5.778	IR
HPRTB	11.2	2	0.042	0	0	6.564	0.01	4.391	3.406	5.661	IR
DXS10148	23	5	0.104	4	0.026	5.367	0.021	4.419	1.137	17.176	IR
DXS10148	18.2	2	0.042	0	0	6.564	0.01	4.391	3.406	5.661	IR
Prostate Cancer (PrCa)											
DXS10079	23	2	0.048	0	0	7.163	0.007	4.714	3.604	6.166	VHR
DXS10148	26	2	0.048	0	0	7.163	0.007	4.714	3.604	6.166	VHR
Both groups (BPH+PrCa)											
DXS10134	39	4	0.044	1	0.006	4.03	0.045*	7.05	0.780	64.02	HR
DXS10134	36.3	3	0.0326	0	0	5.15	0.023	2.753	2.332	3.249	IR
DXS10135	28	3	0.0326	20	0.128	6.29	0.012	0.229	0.066	0.794	PRO
DXS10135	20.1	3	0.0326	0	0	5.15	0.023	2.753	2.332	3.249	IR
DXS10135	20	2	0.0217	16	0.103	5.62	0.018	0.194	0.044	0.866	PRO
DXS10146	38.2	3	0.0326	0	0	5.15	0.023	2.753	2.332	3.249	IR
DXS10079	23	3	0.033	0	0	5.15	0.023	2.75	2.33	3.249	HR
DXS10079	17	9	0.098	5	0.032	4.7	0.03	3.275	1.063	10.092	IR
HPRTB	12.2	3	0.0326	0	0	5.15	0.023	2.753	2.332	3.249	IR
DXS10148	26	3	0.0326	0	0	5.15	0.023	2.753	2.332	3.249	HR
DXS10148	23	8	0.087	4	0.026	4.73	0.03	3.619	1.058	12.375	HR

Sig, Significance; VHR, Very high risk susceptible allele; HR, High risk susceptible allele; IR, Intermediate risk susceptible allele PRO, Protective allele; No., Number of cases; Freq., Frequency of cases; P, P value; OR, Odd Ratio; CI, Confidence Interval, χ2: Chi-Square.

**Table 3:** Description and Function of Genes on X-chromosome which could have Linkage Disequilibrium with the Current Study Significant Haplotypes

Cluster (linkage group)								Gene			Reference
Name	Loci	Haplotype			P- value	Comparison	Location (hg19)	Name	Location (hg19)	Function	
Cluster X1	DXS8378, DXS10135, DXS10148	10	21	23.1	0.001	PrCa>HC	chrX:7,198,150-11,330,000	*TBL1*	chrX:9,621,627-9,684,286	SUMOylation (post-translational modification process) of TBL1 and TBLR1 promotes androgen-independent PrCa cell growth. (SUMO; Small Ubiquitin-like Modifier)	(Park et al., 2016)
		11	30	24.1	0.01	BPH >HC					
Cluster X3	DXS10103, DXS10101, HPRTB	19	32.2	12.2	0.01	BPH>HC	chrX:131,246,000-135,482,000	*ELF4*	chrX:129,198895-129,244688	LOH at this locus identified in ovarian and breast cancers; translocation in acute myelogenous leukemia; rearrangements of BCORL1-ELF4 in hepatocellular carcinomas	(Liu et al., 2012)
		19	30.2	11.2	0.01	BPH >HC		*PHF6 *	chrX:133,507,342-133,562822	Mutation and deletion frequently found in Tcell acute lymphoblastic leukemia, adult acute myeloid leukemia and hepatocellular carcinoma	(Liu et al., 2012)
		17	30	14	0.01	BPH >HC		*LDOC1*	chrX:140,269,931-140,271310	Down regulated in pancreatic and gastric cancer cell lines; deletions identified in PrCa, but high levels of LDOC1 correlate with poor prognosis in chronic lymphocytic leukemia	(Liu et al., 2012)
Cluster X4	DXS10134, DXS7423, DXS10146	35	15	43.2	0.04**	PrCa>HC	chrX:147,310,000-151,460,000	*RPL10*	chrX:153,626571-153,630680	LOH and microsatellite instability at this locus frequently occurred in ovarian cancer; down regulated in PrCa and multiple endocrine neoplasia type 1	(Liu et al., 2012)
		34	14	29	0.01	PrCa>HC		*DKC1 *	chrX:153,991031-154,005964	Sieron et al. did not observe increased gene copy numbers in PrCa with DKC1 overexpression, so they concluded that it likely has an epigenetic role. In this respect, it could be significant that DKC1 was recently identified as a direct target of MYC by Alawi and Lee (2007), a major regulator of cancer cell growth frequently overexpressed in aggressive breast and PrCa.	(Sieron et al., 2009)

PrCa, Prostate Cancer; BPH, Benign Prostate Hyperplasia; HC, Healthy controls, hg19, human genome 19, Microvariant alleles (i.e., 11.2) are designated by the number of full repeats, a decimal, and the number of nucleotides present in the partial repeat (butler, 2011).

## Discussion

We found in our study, that the category of patient who have the intermediate allele have transitional symptoms (BPH) between normal and disease, also they are at risk of developing PrCa; similarly as shown in the study of Savitt and Jankovic (2019) showed that in case of individuals who has intermediate allele of Huntington disease (HD), these individuals and their offspring should be considered at risk for development of progressive HD (Savitt and Jankovic, 2019). Noteworthy that this phenomenon is confirmed by many studies of HD (Apolinário et al., 2017).

During their studies on the STRs instability in mammalian, Suzuki et al. (2001) mentioned that the incomplete repeats found in the microvariant alleles could be the result of DNA polymerase errors during DNA replication.

In a similar way, other STRs, like those analyzed in this study could also affect the gene’s activity. Our hypothesis is that the genetic variants (Alleles) of loci or haplotypes which showed significant differences may be in linkage disequilibrium with tumor-related genes such as oncogenes or tumor suppressor genes. The loci which revealed a microvariant alleles in the BPH group only, DXS7132 (LG1), DXS10146 (LG2), HPRTB (LG3), DXS10134 (LG3) and DXS10135 (LG4) loci are distributed on the X chromosome, at the following regions Xp22.31, Xq11.2-12, Xq26.2 and Xq28, respectively. These loci are located near the *TBL1* gene (promotes androgen-independent PrCa cell growth), *AR* gene (signaling axis plays a key role in androgen- dependent PrCa), *LDOC1* gene (deletions identified in PrCa), and RPL10 (down regulated in PrCa), respectively.

The studies investigated these genes discovered a direct relation to the incidence of PrCa. Post-translational modification process (SUMOylation) of TBL1 and TBLR1 promotes androgen-independent PrCa cell growth. (SUMO; Small Ubiquitin-like Modifier) (Park et al., 2016). *AR* gene is located in Xq12 region and has major roles in proliferation and developments of prostate cells; there were several studies linked between CAG repeats of *AR *gene and BPH risk (Lee et al., 2016). *LDOC1* gene, down regulated in pancreatic and gastric cell lines; deletions identified in PrCa (Liu et al., 2012). *RPL10* gene loss of heterozygosity (LOH) and microsatellite instability at this locus frequently occurred in ovarian cancer; down regulated in PrCa and multiple endocrine neoplasia type1 (Liu et al., 2012). These genes their physical location on 19 human genome and the corresponding haplotypes are listed in Table 3.

A possible genetic susceptibility associated with the abovementioned genes, or another gene within *Xp22.31, Xq11.2-12, Xq26.2* and *Xq28* regions, should be further examined. An important insight into the pathogenesis of BPH/PrCa and other prostate diseases could be provided by this approach which may open this field to promising potential genetic applications.

Some of the microvariant alleles of the above-mentioned loci were already found in some population studies for (German, Hungarian, Malay and four ethnics in the US) population (Becker et al., 2008; Diegoli et al., 2011; Edelmann et al., 2008; Hering et al., 2006; Samejima et al., 2012; Zalán et al., 2007). However, these population samples are not related to Saudi men population and hence do not match our BPH or PrCa cases. Moreover, the individuals who have microvariant alleles in these population studies could have a hidden symptom of BPH or develop it in older age. The most important observation in the current study is that 156 subjects in the control group, their profiles did show any of these microvariant alleles, knowing that inclusion criteria for healthy control group subjects was very restricted to insure that it did not comprise either BPH or PrCa patients. 

Riley and Krieger (2005) studied the STR replacements in untranslated sequences (UTR) among species near disease-related genes, namely in PrCa, and concluded that some STR replacements expand the list of STR sequences that may contribute to genetic activity and to disease process. In a similar way, other STRs, like those analyzed in this study could also affect the gene’s activity. Another possibility is that STR alleles could have themselves a functional effect.

To the best of our knowledge the present study is the first of its kind to analyze 12 X-chromosomal loci in BPH and PrCa patients in the Saudi population and worldwide. Moreover, patients with BPH were also included, in order to find a novel genetic biomarker capable of differentiating between PrCa and BPH, as well as between PrCa and healthy controls. 

The lack of a uniform model of analysis makes the obvious comparison of our findings with previous studies difficult, because most of previous studies used STRs constitute motifs differ from current study, etc., a trinucleotide repeats (CAG, GGC) in the promotor region of androgen receptor (AR), which is located at the Xq11.2–q12 region, an area very close to the loci in cluster 2 in the current study. The CAG repeats normally range from 8 to 35 repeats, with average 20 repeats. As per Gu et al., (2012), meta-analysis results showed that CAG repeat polymorphism on *AR *gene with ≥ 20 repeats might confer a protective effect among 45 year-old patients with PrCa but not all patients with PrCa; moreover, Alptekin et al., (2012) and Lee et al., (2016) also reported that short repeats may comprise high risk for PrCa and BPH, respectively. The other GGC trinucleotide repeats range from 10 to 30; the effect of the variation in the length of the GGC tract on AR activity is unclear, but it has been thought that it has a role in the transcriptional activity of the *AR* gene, but the repeat did not show any significant frequency differences between patients with BPH or PrCa and healthy controls (Alptekin et al., 2012).

We could not find a study investigate the association between STRs on ChrX regions other than Xq11.2–q12 region and BPH and PrCa; meanwhile, Gu et al., (2012) suggested to investigate *AR* adjacent genetic markers to confirm whether the present association is causal or due to LD. Furthermore, upon their observations, Riley and Krieger (2002) have been suggested that Xq11–13 region may contain one or more genetic loci that predispose toward chronic prostatitis. They also suggested that STRs in the Xq11–Xq13 region and other regions may provide a means to rapidly and comprehensively scan genetic loci in large populations of patients with PrCa and healthy controls (Riley and Krieger, 2001)

In the comparison of haplotypes among current study groups, the results did not show significant differences, these findings are consistent with two studies which investigated the association between AR CAG repeat length and PrCa; however, they found a relation between short CAG repeat and high grade and advanced stage of disease; also a relation with PrCa patients diagnosed at young age (Bratt et al., 1999; Giovannucci et al., 1997).

These findings support our hypothesis that ChrX has a role in susceptibility or resistance to BPH and PrCa, the benefit of STRs that has LD with certain genes responsible for development or resistant to PrCa, in scanning chromosomes to find association between alleles and BPH or PrCa. Another study investigated CAG polymorphism as a categorical variable and BPH risk in the Brazilian population, but found no evidence for association (Biolchi et al., 2012); this lack of relation may be related to different cut off point used. Moreover, it is a fact that the contribution of genetic polymorphisms to the risk of PrCa or BPH may be dependent on the population under study, as well on several environmental and diet factors that influence the population (Medeiros et al., 2003).

In conclusion, the results of this study are in concordance with the involvement of X-chromosome in BPH development. X-chromosome STRs may be valuable in determining susceptibility profiles. the combined typing of multiple STRs may provide practical diagnosis of a patient’s vulnerability to BPH or PrCa. These unexpected findings suggest that the intermediate-risk category is a crucial cohort warranting further study to determine if a unique molecular fingerprint can predict aggressive versus indolent phenotypes. Finally, to scan the X-chromosome with other polymorphic markers could provide possible evidence to use such genetic information to predict a man’s predisposition to prostatic diseases or his response to medical interventions.

## List of Abbreviations

PrCa, Prostate cancer; BPH, Benign Prostate Hyperplasia; STRs, short tandem repeats; GD, Genetic Diversity; PSA, prostate-specific antigen; LD, Linkage Disequilibrium.
